# Standard reference values of the upper body posture in healthy male adults aged between 51 and 60 years in Germany

**DOI:** 10.1038/s41598-022-10917-2

**Published:** 2022-04-28

**Authors:** Daniela Ohlendorf, Dominik Krüger, Wolfgang Christian, Hanns Ackermann, Fee Keil, Gerhard Oremek, Christian Maurer-Grubinger, David A. Groneberg

**Affiliations:** 1 Institute of Occupational, Social and Environmental Medicine, Goethe-University Frankfurt/Main, Theodor-Stern-Kai 7, Building 9a, 60596 Frankfurt/Main, Germany; 2 Institute of Biostatistics and Mathematical Modeling, Goethe-University, Frankfurt/Main, Theodor-Stern-Kai 7, Building 11A, 60596 Frankfurt/Main, Germany; 3 Institute of Neuroradiology, Goethe-University, Frankfurt/Main, Theodor-Stern-Kai 7, Building 95, 60596 Frankfurt/Main, Germany

**Keywords:** Orthopaedics, Translational research

## Abstract

Comparative values are essential for the classification of orthopedic abnormalities and the assessment of a necessary therapy. At present, reference values for the upper body posture for healthy, male adults exist for the age groups of 18–35, 31–40 and 41–50 years. However, corresponding data on the decade of 51 to 60 year-old healthy men are still lacking. 23 parameters of the upper body posture were analyzed in 102 healthy male participants aged 51–60 (55.36 ± 2.78) years. The average height was 180.76 ± 7.81 cm with a weight of 88.22 ± 14.57 kg. The calculated BMI was 26.96 ± 3.92 kg/m^2^. In the habitual, upright position, the bare upper body was scanned three-dimensionally using video raster stereography. Mean or median values, confidence intervals, tolerance ranges and group comparisons, as well as correlations of BMI and physical activity, were calculated for all parameters. The spinal column parameters exhibited a good exploration of the frontal plane in the habitual standing position. In the sagittal plane, a slight, ventral inclination of the trunk with an increased kyphosis angle of the thoracic spine and increased thoracic bending angle was observed. The parameters of the pelvis showed a pronounced symmetry with deviations from the 0° axis within the measurement error margin of 1 mm/1°. The scapula height together with the scapula angles of the right and left side described a slightly elevated position of the left shoulder compared to the right side. The upper body posture is influenced by parameters of age, height, weight and BMI. Primarily there are significant correlations to measurements of trunk lengths D (age: p ≤ 0.02, rho = -0.23; height: p ≤ 0.001, rho = 0.58; weight: p ≤ 0.001, rho = 0.33), trunk lengths S (age: p ≤ 0.01, rho = -0.27; height: p ≤ 0.001, rho = 0.58; weight: p ≤ 0.001, rho = 0.32), pelvic distance (height: p ≤ 0.01, rho = 0.26; weight: p ≤ 0.001, rho = 0.32; BMI: p ≤ 0.03, rho = 0.22) and scapula distance (weight: p ≤ 0.001, rho = .32; BMI: p ≤ 0.01, rho = 0.27), but also to sagittal parameters of trunk decline (weight: p ≤ 0.001, rho = -0.29; BMI: p ≤ 0.01, rho = -0.24), thoracic bending angle (height: p ≤ 0.01, rho = 0.27) and kyphosis angle (BMI: p ≤ 0.03, rho = 0.21). The upper body posture of healthy men between the ages of 51 and 60 years was axially almost aligned and balanced. With the findings of this investigation and the reference values obtained, suitable comparative values for use in clinical practice and for further scientific studies with the same experimental set-up have been established.

## Introduction

Demographic change reveals that the average age of the population continues to rise and that the proportion of older generations in relation to the younger ones^1,2^ is increasing due to improved medical care^2^, an increasing life expectancy^3^ and a declining birth rate^4^. The world of labor is subject to a similar development; the retirement age is being raised further and the possibility, even for chronically ill persons, to apply for an early retirement at the age of 55 years is becoming much more difficult^5,6^. The average age of salaried assembly line workers in Germany is now 50 years, compared to 30 years only 20 years ago^6^. According to a study for the Federal Statistical Office by Booz & Company^7^, 40% of the workforce in Germany will be between 50 and 65 years old by 2024. The combination of aging and prolonged work phase, with the accompanying increased physical and mental stress experienced by the older person, will prognostically increase the occurrence of certain diseases^8^; these include, above all, mental illnesses, cardiac insufficiencies, joint degeneration and back problems^9^. In Germany, the most recent 2020 health surveys found that 66% of women and 56.4% of men suffer from back pain, of which 23.2% complain of severe pain and 6.4% even report significant impairment due to back pain^10,11^. One 2020 health survey^10^ states that men in the age decade of 50–59 years complain most frequently of back pain at 63% and, furthermore, the largest proportion of patients with very severe complaints is also within this age group for both sexes. In this context, the increasing digitalization and tertiarization of the work sector must also be taken into account. With the shift of sedentary work in a desk environment, with air conditioning, unnatural light and reduced physical challenges may develop musculoskeletal complaints^12,13^. Work-related psychosocial stress, which is characterized by high workload, low social support, low job satisfaction and monotonous work, also harbors a potential risk of musculoskeletal disorders^13^.

Fundamentally, posture is a multifactorial construct, including internal^14^, hormonal^15^ and psychological^16^ factors that can cause change. This complex concatenation demonstrates the difficulty of making a reliable diagnosis. Thus, a detailed anamnesis is the basis of any diagnosis, followed by an examination of the patient, possibly supported by technical aids and laboratory findings. For the evaluation of these findings, it is necessary to have comparable reference values available in order to classify corresponding deviations and to be able to draw therapeutic conclusions. Accordingly, it makes sense to determine the corresponding standard values of the upper body statics in an unstressed group of people in order to compare these with pathological findings and, thus, aid in confirming the diagnosis. Data on posture in selected subject groups, such as mentally ill persons^17^, scoliosis patients^18^, male and female children aged 7–9 years^19^ and men and women aged over 60 years (n = 70)^20^, do exist. The latter authors were able to prove that males have a significantly increased lumbar lordosis angle compared to females of the same age. In a second paper^21^, the focus was limited to a comparison of 130 female participants, between 60 and 90 years of age, with 130 female young adults between 20 and 25 years of age using the same study design. It was found that the older the subjects were, the more forward tilt of the upper body occurred, with the gamma angle of the thoracic spine increasing significantly from 38.42° in 60–70 year-olds to 54.28° in the 81–90 year-old age group. The thoracic depth increased by 26.69 mm between the same age decades (mean 60–70 years: − 0.47 mm; mean 81–90 years: − 27.16 mm). Moreover, the asymmetry of the shoulder position increased with age. Similar results were obtained by the photometric study of Gong et al.^22^; the greatest age-related deviations were found in the increased curvature of the cervical and thoracic spine from the age of 50 years. In addition, Grabara et al.^23,24^ recorded sport-specific characteristics of the upper body posture.

The major project of Ohlendorf’s research group has previously determined age- and gender-specific reference values of the dorsal upper body posture in subjectively healthy participants^25^. Therefore, within this research group, there already exists relevant comparative values of the back parameters for male subjects between the ages of 18 and 35 years^26^, 31–40 years^27^ and 41 to 50 years^28^ and for females between the ages of 21–30 years^29^ and 51–60 years^30^. Thus, the present study, as a component of the research group, sought to present appropriate evaluations for a male subject population aged 51–60 years.

The study's participants were male and between the ages of 51 to 60 years; this meant that they had been in the workforce for a long time, the body was no longer as adaptable^31,32^ and that physical changes could be detected^33,34^.

Hence, this study would provide a better understanding of the upper body posture of a healthy male in this age group in relation to these variables and would present the corresponding comparative values to existing pathologies.

## Material and methods

This study is part of a major project^25^, parts of which, related to the standard values of upper body posture, have already been published^27,30,35–37^.

### Subjects

The present study examined 102 subjectively healthy men aged 51 to 60 years (55.36 ± 2.78 years) with a height of 180.76 ± 7.81 cm and a weight of 88.22 ± 14.57 kg. The resulting body mass index (BMI) ranged from 20.76 to 39.68 kg/m^2^ (26.96 ± 3.92 kg/m^2^). Based on the BMI classification according to the WHO criteria^38^, 38 subjects (37.25%) were, thus, classified as normal weight (BMI 18.5–24.9 kg/m^2^), 46 (45.10%) were considered preadipose (BMI 25–29.9 kg/m^2^) and 18 participants (17.65%) were counted as being obese (BMI ≥ 30 kg/m^2^). Four of the obese subjects were found to have second-degree obesity (BMI 35–39.99 kg/m^2^) according to the definition^38^, but this was not further subdivided in this study. Underweight subjects (BMI < 18.5 kg/m^2^) were not present among the subject collective. Concerning handedness, 86.28% (n = 88) of the men were right-handed, 9.80% were left-handed (n = 10) and 3.92% (n = 4) were ambidextrous.

Regarding sports activity, 27.45% rarely to never exercised, 28.43% exercised once or twice a month, 19.61% exercised once a week, 16.67% exercised regularly twice a week, while 7.84% exercised at least three times a week. Accordingly, various occupational groups of different daily workloads, which ranged from software developers to postal workers to employees in nursing services, were integrated and, thus, represented a broad cross-section of the population.

In terms of occupational stress, 45.10% of the study participants reported having a sedentary job, 20.59% were more likely to have a varied job and 29.41% were more severely exposed to physical stress through work, however, 4.90% did not provide any information on this subject.

Participation in the study required the subject to be free from complaints, which inferred unrestricted mobility and freedom from complaints regarding the musculoskeletal system. Furthermore, the absence of current discomfort, pain or disease of the spine, musculoskeletal system and temporomandibular system, and the absence of surgery/injury within the past two years in these areas were required for participation. Finally, the subjects were required neither to be undertaking physical therapy nor orthopedic treatment and not to be using muscle relaxants or have any physician-diagnosed physical deformities at the time of the study. No radiation-based measurements, such as X-ray or CT, were performed for the status of the spine to confirm the health status of the subjective subject statement. Non necessary radiation is considered unethical in Germany. In addition, other medical history information was collected, such as information on headaches/migraines, temporomandibular joint disorders, orthodontic treatment, handedness, age, height and weight. For this purpose, a modified medical history form of the Dental University Institute of the Johann Wolfgang Goethe University Frankfurt am Main was used^39^.

An approved ethics application from the Department of Medicine of the Johann Wolfgang Goethe University has been submitted for the performance of this study (Ethics No. 103/16). All methods were performed in accordance with the relevant guidelines and regulations" (e.g. Declaration of Helsinki).

### Three-dimensional back measurement

The "Back Mapper Mobile" back scanner (ABW GmbH, Frickenhausen, Germany) was used in this study. This measures at a maximum frame rate of 50 frames per second with a depth resolution of 1/100 mm. According to the manufacturer, it has a feature accuracy of < 1 mm and a feature repeatability of < 0.5 mm. According to the manufacturer, the accuracy of the marker placement is less than one millimeter. It should be noted that the feature accuracy is synonymous with the accuracy of the measured parameters, however, the accuracy of the marker placement is not the same as the operator-dependent reproducibility (see limitations). Specifying the decimal places in the results does not increase the accuracy of the measurement. Usually, rounded integers are given. However, it was used this way for better statistical analysis.

The scanner unit contains the light projector which casts the strip grid onto the object, and an LED camera which detects the line curvature image. Using the triangulation technique, the upper body posture can, thus, be displayed in three dimensions, divided into three areas (spine, pelvic girdle and shoulder girdle). Overall, this is a radiation-free and non-invasive measurement method.

Studies, e.g., by Liljenqvist et al.^40^, Drerup et al.^41^ and Schroeder et al.^42^ show that video raster stereography as a noncontact, three-dimensional surface measurement is a reliable method for measuring the spine and is capable of validly reproducing isolated radiographic parameters.

### Examination procedure

**Figure 1:**
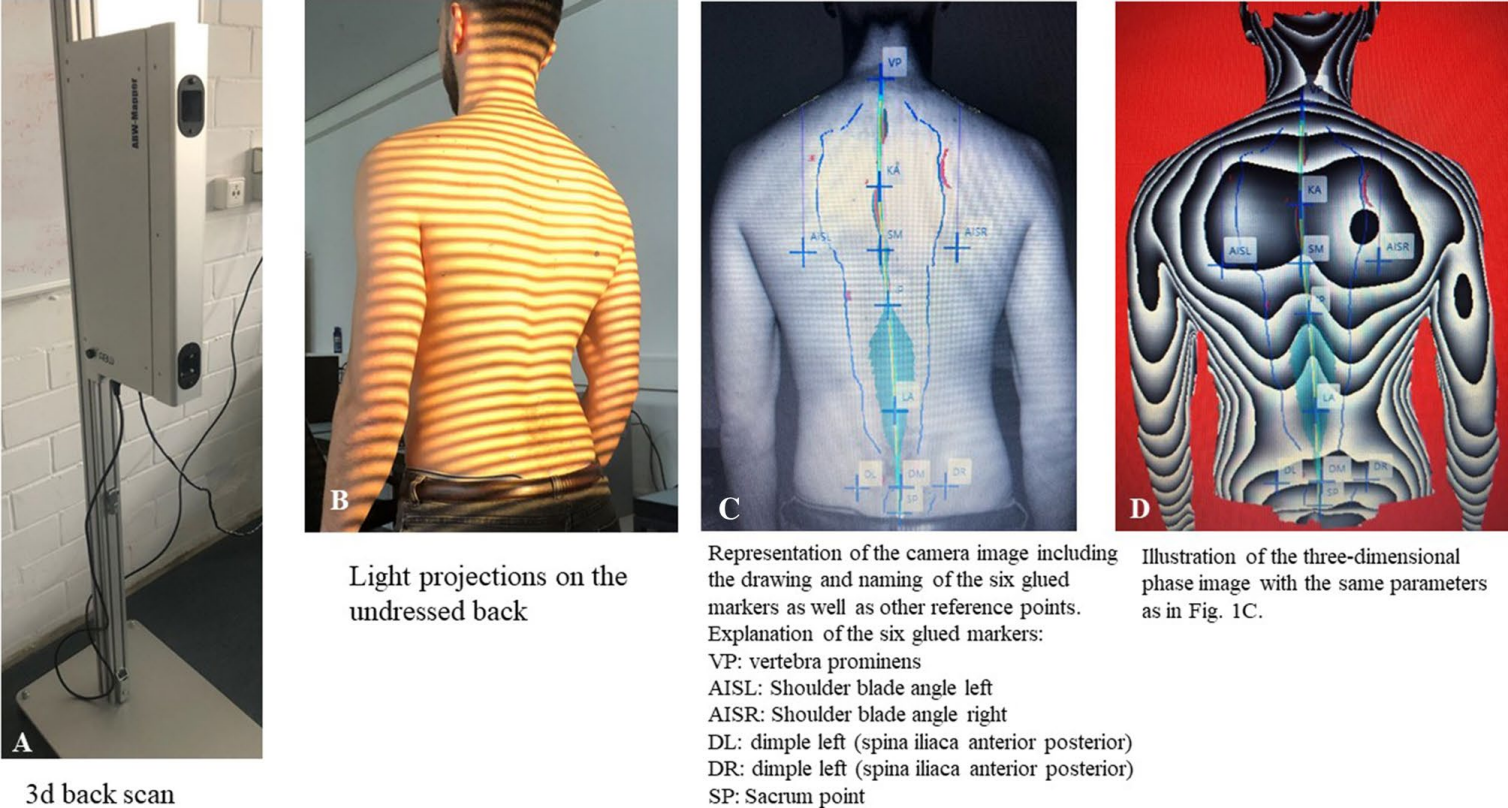
Marking of the six anatomical fixed points. These points comprised the vertebra prominens (cervical vertebra C7), the right and left angulus inferior scapulae, the right and left spina iliaca posterior superior and the most cranial point of the rima ani.

Prior to the start of the measurements, each participant had six anatomical reflective markers taped onto their bare back; these were necessary for the software to calculate the parameters. These six reference points included the vertebra prominens (cervical vertebra C7), the right and left angulus inferior scapulae, the right and left spina iliaca posterior superior and the most cranial point of the rima ani (Fig. 1).

For the measurements, the subject stood at about 90 cm in front of the back scanner with a shoulder-width stance in an upright, habitual posture with a gaze directed freely straight ahead and the arms hanging relaxed beside the body. The axial alignment of the test person is ensured by a template which, starting from the scanner's stool, on the one hand ensures the exact distance between the subject and the scanner and, on the other hand, a bar is attached to the other end which the heels of the test person to be measured touch. The measurements were repeated three times to statistically reduce the postural sway.

#### Evaluation parameters

During the optical measurement of the body statics, further relevant points were determined by the associated software with the aid of the six marked reference points. Based on these points and the lines formed from them, the software of the ABW Mapper calculated a total of 23 distances and angles of the spine (13), pelvis (5) and shoulder girdle (5) in all three planes. An explanation of all the parameters can be found in Ohlendorf et al.^25^ and in Table 1.

**Table 1:** Representation of the median/mean values, the tolerance ranges with lower and upper limits and the confidence intervals with left and right limits for all back parameters.

	Mean value/median	Tolerance range lower limit	Tolerance range upper limit	Confidence interval left limit	Confidence interval right limit
**Spine parameter**					
Trunk length D (mm) Spatial distance between the markers C7 and middle of the PSIS marker	497.55	438.73	556.37	491.75	503.35
Trunk length S (mm) Spatial distance between the markers at C7 and rima ani	536.26	475.19	597.34	530.25	542.28
Sagittal trunk decline (°) Inclination of the trunk length D marked line from the perpendicular to the sagittal plane	− 3.71	− 9.49	2.07	− 4.28	− 3.14
Frontal trunk decline (°) Inclination of the trunk length D marked line from the perpendicular to the frontal plane	− 0.35	− 3.25	2.55	− 0.63	− 0.06
Axis decline (°) Deviation of the line of the area marked by the trunk length D line of the 90° rotated distance between PSIS left and PSIS right	− 0.31	− 5.21	4.58	− 0.80	0.17
Thoracic bending angle (°) Deviation of the distance C7 – kyphosis apex from the perpendicular	17.43	9.26	25.61	16.63	18.24
Lumbar bending angle (°) Deviation of the distance kyphosis apex—lordosis apex from the perpendicular	10.82	4.82	16.83	10.23	11.42
Standard deviation lateral deviation (°) Root mean squared deviation of the median line of the distance C7—center of the PSIS marker	3.76	1.11	9.34	3.22	4.40
Standard deviation rotation (°) Root mean square deviation of surface rotation of the median line (torsion of the spinous processes of the spine)	3.90	1.47	10.64	3.46	4.56
Kyphosis angle (°) Angle between the upper turning point at C7 and the thoracolumbar inflection point	53.99	36.99	70.98	52.31	55.66
Lordosis angle (°) Angle between the lower inflection point at the center of the PSIS marker and the thoracolumbar turning point	33.40	14.05	52.75	31.50	35.31
**Shoulder parameter**					
Scapular distance (mm) Distance between the left (AISL) and the lower right (AISR) scapular angles	197.70	153.61	241.79	193.36	202.04
Scapular height (°) Height difference between the AISL and AISR points	− 0.37	− 22.61	23.30	− 2.16	2.30
Scapular rotation (°) Rotation of the distance AISL—AISR in the transversal plane	1.15	− 8.53	7.42	0.57	2.14
Left scapular angle (°) Angle of the compensation line applied from the shoulders to the horizontal. The center of the compensation line is specified vertically above AISL	26.13	15.55	36.70	25.09	27.17
Right scapular angle (°) Angle of the compensation line applied from the shoulders to the horizontal. The center of the compensation line is specified vertically above AISR	28.21	19.08	54.85	26.49	29.48
**Pelvis parameter**					
Pelvic distance (mm) Spatial distance between the left (PSISL) and right (PSISR) of the pelvis	93.85	66.43	121.28	91.15	96.56
Pelvic height (°) Decline of the connecting line between the PSIS left and PSIS right to the horizontal in the frontal plane in degrees	− 0.01	− 4.33	4.31	− 0.43	0.42
Pelvic height (mm) Decline of the connecting line between the PSIS left and PSIS right to the horizontal in the frontal plane in millimeters	0.07	− 6.89	7.04	− 0.61	0.76
Pelvic torsion (°) PSIS left—PSIS right, twist around the transverse axis calculated from the mutual twisting of the surface normal on the two PSIS	− 0.20	− 11.12	10.72	− 1.28	0.88
Pelvic rotation (°) Rotation of the distance PSIS left—PSIS right in the transversal plane	− 0.71	− 8.13	6.71	− 1.44	0.02

### Statistical evaluation

The statistical evaluations of the collected measurement results were carried out with the software program BIAS, version 11.10 (epsilon-Verlag GbR, Darmstadt). In order to be able to make the selection for the suitable test procedures, the normal distribution for each measurement parameter was checked by means of the Kolmogoroff-Smirnoff-Lilliefors test. Accordingly, parametric or non-parametric tests were used. For the descriptive statistics, mean or median values, either with the corresponding standard deviation or the 1st and 3rd quartiles, were determined as well as the tolerance range (TR) with its lower (loL) and upper limits (uL) and the confidence interval (CI) with the left (leL) and right limits (rL).

Tolerance regions were defined by the upper and lower limit for 95% of all values (= ± 2σ values), which have been found in about 95% of the examined subjects. Here, all values have to be considered as normal. Consequently, the tolerance range estimates the central part of 95% of the value of the measured subject population. The two-sided 95% CI indicates the possible range for the mean or median value depending on the distribution quality. It shows the ‘accuracy’ of these values.For a group comparison of more than two groups, the Kruskal–Wallis test for non-normally distributed data was used followed by the Conover-Iman test including Bonferroni-Holm correction. The effect size "eta^2^" was set according to Rasch (eta^2^ = 0.01 small effect, eta^2^ = 0.06 medium effect, eta^2^ = 0.14 large effect).

With regard to the correlation calculations, the rank correlation according to Spearman or Pearson was used. The correlation coefficient rho was evaluated in its effect strength according to Evans (rho < 0.2 very weak, rho = 0.2–0.4 weak, rho = 0.4–0.6 moderate, rho = 0.6–0.8 strong, rho > 0.8 optimal). The significance level was set at 5%.

### Ethics approval and consent to participate

This study was approved by the Ethics Committee of the Department of Medicine of the University Hospital of the Goethe University Frankfurt am Main (Number: 103/16). All participants signed an informed consent to participate in advance, so the consent was written. Minors were excluded as participants of this study.

## Results

Table 1 lists the median and mean values with the corresponding tolerance ranges (TR) and confidence intervals (CI) obtained in this study.

The trunk length D had a mean value of 497.55 mm (. The trunk length S was approximately 40 mm longer and averaged 536.26 mm. The upper body inclination of the subjects was, on average, − 3.71° ventrally inclined. In the frontal plane, the trunk deviated an average of 0.35° to the left. The axial deviation of the spine to the pelvis, with a mean value of − 0.31°, also indicated a slight leftward tilt of the upper body.

The thoracic bending angle with its mean value of 17.43° described a stronger kyphosis of the cranial thoracic section compared to the caudal section, which was characterized by the lumbar bending angle with a mean value of 10.82°. The kyphosis angle of the thoracic spine averaged 53.99° in the subjects. The lordosis angle of the lumbar spine was 33.40° on average. The spine was tilted to the right by an average of 3.76 mm and also rotated in the transverse plane by 3.90°.

The measurement parameters of the basin showed a mean value of the basin distance of 93.85 mm. The mean basin level was nearly horizontal at − 0.01° and 0.07 mm, respectively. Here, positive values described a higher pelvic level on the right side; a negative sign indicated an increased pelvic level on the left side. The pelvic torsion describes the twisting of the pelvic blades in relation to each other and was indicated with a mean value of − 0.20° in the test subjects. Accordingly, the left side of the pelvis was, on average, minimally more upwardly directed. The pelvic rotation was − 0.71°, after which there was a slight, right-sided rotation in which the right side of the pelvis was more anterior.

The mean scapula distance was 197.70 mm. In addition, the scapula was minimally more cranial on the left (− 0.37 mm) and tended to be rotated 1.15° dorsally on the right. Overall, the left shoulder was minimally more cranial. Here, the median value of the left shoulder stance angle was 26.13° and the median value of the right shoulder stance angle was 28.21°.

### Analysis of constitutional and anamnestic parameters

Table 2 presents the data on the correlation between the age, height, weight and calculated BMI versus the back parameters.

**Table 2:** Presentation of the p-values with associated correlation coefficients.

Parameter	Age		Height		Weight		BMI	
	p-value	rho	p-value	rho	p-value	rho	p-value	rho
**Spine parameter**								
Trunk length D (mm)	**0.02**	− 0.23	**0.001**	0.51	**0.001**	0.33	0.33	0.10
Trunk length S (mm)	**0.01**	− 0.27	**0.001**	0.58	**0.001**	0.32	0.57	0.06
Sagittal trunk decline (°)	0.71	− 0.04	0.07	− 0.18	**0.001**	− 0.29	**0.01**	− 0.24
Frontal trunk decline (°)	0.99	0.001	0.07	− 0.18	0.59	0.05	0.09	0.17
Axis decline (°)	0.54	− 0.06	0.96	0.01	0.51	0.07	0.52	0.06
Thoracic bending angle (°)	0.35	− 0.09	**0.01**	0.27	0.19	0.13	0.98	0.01
Lumbar bending angle (°)	0.87	− 0.02	0.54	− 0.06	0.15	− 0.14	0.19	− 0.13
Standard deviation lateral deviation (°)	0.22	0.12	0.19	0.13	0.5	0.07	0.87	0.02
Standard deviation rotation (°)	0.34	− 0.09	0.62	0.05	0.80	− 0.02	0.68	− 0.04
Kyphosis angle (°)	0.62	0.05	0.99	− 0.001	0.08	0.17	**0.03**	0.21
Lordosis angle (°)	0.31	0.10	0.08	− 0.17	0.17	− 0.14	0.51	− 0.07
**Pelvis parameter**								
Pelvic distance (mm)	0.32	0.10	**0.01**	0.26	**0.001**	0.32	**0.03**	0.22
Pelvic height (°)	0.60	− 0.04	0.29	0.11	0.59	0.05	0.91	− 0.01
Pelvic height (mm)	0.62	− 0.05	0.30	0.10	0.54	0.06	0.99	0.01
Pelvic torsion (°)	0.15	0.14	0.25	− 0.11	0.54	− 0.06	0.90	− 0.01
Pelvic rotation (°)	0.91	− 0.01	0.84	− 0.02	0.90	0.01	0.82	0.02
**Shoulder parameter**								
Scapular distance (mm)	0.93	0.01	0.22	0.12	**0.001**	0.32	**0.01**	0.27
	0.72	− 0.04	0.37	− 0.09	0.94	− 0.01	0.95	0.01
Scapular height (°)	0.71	0.04	0.94	0.01	0.87	− 0.02	0.67	− 0.04
Scapular rotation (°)	0.52	− 0.07	0.96	− 0.01	0.79	− 0.03	0.52	− 0.06
Left scapular angle (°)	0.91	− 0.01	0.39	0.09	0.46	− 0.07	0.12	− 0.16
	0.93	0.01	0.22	0.12	**0.001**	0.32	**0.01**	0.27
Right scapular angle (°)	0.72	− 0.04	0.37	− 0.09	0.94	− 0.01	0.95	0.01

Correlation coefficients are according to Evans [rho < 0.2 very weak, rho = 0.2–0.4 weak, rho = 0.4–0.6 moderate, rho = 0.6–0.8 strong, rho > 0.8 optimal] when comparing the back parameters to the parameters of age, height, weight and BMI. Significant p-values are printed in bold.

The subject age showed a negative correlation to the trunk length D and trunk length S indicating that the trunk length reduced with increasing age.

Furthermore, a positive, significant correlation between body size and trunk length D as well as S, the thoracic bending angle and pelvic distance could be observed. In addition, correlations of body size to thus, the taller the participants, the more curved is the upper thoracic segment and the wider the pelvic distance.

There were also significant correlations with p-values of ≤ 0.001 between weight and the trunk length D, trunk length S, pelvic distance, scapula stance and sagittal trunk tilt. This means that with increasing weight, the participants were found to be taller and wider in the pelvic and scapular distance, respectively, with the trunk tilting further ventrally the heavier the participant.

The BMI was also statistically related to sagittal trunk decline, pelvic and scapula stance. As well as the kyphosis angle, such that the kyphosis angle of the thoracic spine increased the heavier the subjects were.

All other correlations were not statistically significant.

For the grouping of the BMI according to the WHO criteria (normal weight, preadipose, obese), the group comparisons per back parameter yielded a statistical significance only for the kyphosis angle (p ≤ 0.05, eta^2^ = 0.06). In the subsequent multiple Conover-Iman comparisons, there were no significant pairwise comparisons following Bonferroni-Holm correction.

### Sports activity

The data of the subjects' sports activity were divided into four relevant group sizes (1: rarely/never, 2: once a month, 3: once a week, 4: twice a week) and compared with each other (group 5, due to too small a group size, was excluded from the analysis). A significance in the pelvic level (°) (p ≤ 0.5; eta^2^ = 0.09) was no longer supported following a Conover-Iman comparison including Bonferroni-Holm correction. All other comparisons were not statistically significant.

## Discussion

With an average height of 180.76 cm, this study's subjects are almost the same height as younger men in previous studies^27,35,37^. Furthermore, the average weight (88.22 kg) of the subjects and their calculated BMI (26.96 kg/m^2^) differ little from those in the study of 41- 50 year-old men (weight: 88.76 kg; BMI: 26.02 kg/m^2^)^37^. However, in previous studies, younger subjects (aged 18 to 35 years) were significantly lighter (77.20 kg) than those of the present study and also had a lower BMI (23.60 kg/m^2^)^35^, while the parameters of men aged 31–40 years were close to those of the present group with their weight (85 kg) and calculated BMI (26.20 kg/m^2^)^27^. Nevertheless, the values of all age groups correlate with the current data of the Federal Statistical Office^43^. Only from the age of 60 to 65 years do the height and weight, with a relatively constant BMI, decrease again at an increasing rate^43,44^.

In contrast, the upper body posture in the present collective is almost symmetrical in the frontal plane, with a slightly ventrally directed upper body. Furthermore, there is a rightward curvature of the spine (3.76 mm) with the vertebral bodies rotated to the right (3.90°). Measured from the apex of the thoracic spine, the thoracic bending angle averaged 17.43°; this is 6.61° greater than the lumbar bending angle (10.82°) indicating a greater kyphosis of the thoracic spine with a propulsed head posture. A significantly larger kyphosis angle of 53.99° compared to the lordosis angle of 33.40° confirms these data.

The pelvis (pelvic distance: 93.85 mm) is nearly symmetrical and balanced, as is the shoulder area (scapula distance 197.70 mm). However, the right shoulder is minimally more cranial than the left, with little dorsal right rotation; this could be related to the general rotation of the spine to the right. Overall, the participating subjects exhibit a relatively symmetrical, plumbed posture as several measurement data fall within the ± 1°/1 mm measurement error range.

The present data can be classified according to the pre-existing data of the whole project^25^ concerning the reference values of the upper body posture of male subjects aged 18–35 years^35^, 31–40 years^27^ and 41–50 years^37^. A comparison of the present study's male reference values of constitutional parameters and back parameters with other age groups of this project is shown in Table 3.

**Table 3:** Comparison of the median and mean values of the constitutional parameters and back parameters of different male age groups from the research of Ohlendorf et al.

Parameter	Median/mean				
	Men 18–35 years^35^	Men 31–40 years^27^	Men 41–50 years^37^	Men 51–60 years	Max. deviation among men
**Constitutional parameter**					
Height [cm]	181.00	179.89	181.00	180.76	1.11
Weigth [kg]	77.20	85.00	88.76	88.22	11.56
BMI [kg/m^2^]	23.60	26.20	26.20	26.96	3.36
**Back scan parameter**					
Trunk length D (mm)	478.42	473.95	500.17	497.55	26.22
Trunk length S (mm)	528.44	526.03	543.76	536.26	15.32
Sagittal trunk decline (°)	− 3.62	− 2.63	− 3.40	− 3.71	0.77
Frontal trunk decline (°)	0.33	− 0.34	− 0.30	− 0.35	0.68
Axis decline (°)	− 0.34	− 0.98	− 0.83	− 0.31	0.67
Thoracic bending angle (°)	16.34	15.66	15.76	17.43	2.77
Lumbar bending angle (°)	10.10	10.99	10.34	10.82	0.89
Standard deviation lateral deviation (°)	3.83	4.70	3.50	3.76	1.20
Standard deviation rotation (°)	3.67	3.41	3.71	3.90	0.49
Kyphosis angle (°)	45.85	52.56	51.08	53.99	8.14
Lordosis angle (°)	30.67	32.16	32.86	33.40	2.73
Pelvic distance (mm)	93.68	127.42	92.84	93.85	34.58
Pelvic height (°)	− 0.77	− 0.46	− 0.50	− 0.01	0.76
Pelvic height (mm)	− 1.24	− 1.25	− 1.13	0.07	1.32
Pelvic torsion (°)	− 0.43	1.16	0.12	− 0.20	1.59
Pelvic rotation (°)	− 0.86	0.32	0.19	− 0.71	1.03
Scapular distance (mm)	179.23	185.45	186.04	197.70	18.47
Scapular height (°)	− 2.45	− 4.35	− 0.89	− 0.37	3.98
Scapular rotation (°)	0.52	0.77	1.65	1.15	1.13
Left scapular angle (°)	26.00	26.60	26.74	26.13	0.74
Right scapular angle (°)	29.00	27.93	28.49	28.21	1.07

Values are taken from this work and research papers^27,35,37^.

The most noticeable changes are in the kyphosis and lordosis angles. Both angles increase with age, with the kyphosis angle showing an overall increase of 8.14° and the lordosis angle increasing somewhat more weakly by 2.73° compared to the 18–35 year-old male age group^35^. Similarly, matching the effect of age on the kyphosis angle is the increase of the thoracic bending angle by 2.67° when compared to that of the 41–50 year-old men^37^. The greater curvature of the lumbar lordosis can be explained by the increasing body weight or BMI in that possibly the weight of the abdominal fat causes ventral traction^45^. In addition, there is a decrease in muscle mass with increasing age and associated body tension^46,47^ which favors a protracted shoulder position. Less than 45% of the present study's cohort exercised at least once a week and, furthermore, 45.10% of the subjects had a sedentary office job. These characteristics mainly favor the decrease of musculature in the scapula region and a decreasing strength for straightening of the back^48–51^. This reasoning also supports the observation of the increasing scapula distance with age, which steadily increases from 179.23 mm (18–35 year-olds^35^) to 197.70 mm (51–60 year-olds). For the other parameters, the differences are within the range of the measurement error and do not indicate any relevant, age-specific changes.

When looking at the results for the female subjects of the same age^30^ in this series of examinations, summarized in Table 4, it can be seen that the largest gender-specific differences exist for the lordosis and kyphosis angles. The lordosis angle of the female subjects is 52.61°, which is a difference of 19.21° of increased lumbar lordosis compared to the median value of the males (33.40°). The kyphosis angle of females is also more pronounced, but only with a difference of 6.50° (median males: 53.99°; median females: 60.49°). This shows that the curvature of the spine of the female test subjects has a clearly pronounced amplitude overall. In contrast, the spine of male participants stands somewhat straighter, which can be explained by the anatomical differences between the sexes^52^. The female pelvis shows a different shape and position to the surrounding joints^53^. The sacrum is anatomically located between the pelvic scoops with a steeper angle of 60° in females, whereas the pelvic tilt angle is lower at 50–55° in males^54,55^. This inclination continues to the lumbo-sacral junction and, thus, provides for the stronger lordosis position in the lumbar region^56–58^ which is compensatorily accompanied by a likewise, more pronounced oscillation of the thoracic spine kyphosis^59^. Further deviations from the normal values of the back parameters from the results of the corresponding female age group^30^ are found for the trunk lengths D and S with 45.23 mm and 48.64 mm, respectively, as well as a difference in the scapula distance (33.64 mm). These differences can be explained by the different anatomical conditions. All other parameters show no gender-specific abnormalities. Accordingly, age- and sex-specific deviations of the normal values can be described.

**Table 4:** Comparison of the median and mean values of the constitutional parameters and back parameters of males and females in the age group 51 to 60 years from the research of Ohlendorf et al.

Parameter	Median/mean		
	Men 51–60 years	Women 51–60 years^30^	Maximum deviation between men/women 51–60 years
**Constitutional parameter**			
Height [cm]	180.76	166.00	24.76
Weigth [kg]	88.22	69.30	18.92
BMI [kg/m^2^]	26.96	25.02	1.94
**Back scan parameter**			
Trunk length D (mm)	497.55	452.32	45.23
Trunk length S (mm)	536.26	487.62	48.64
Sagittal trunk decline (°)	− 3.71	− 3.95	0.24
Frontal trunk decline (°)	− 0.35	− 0.31	0.04
Axis decline (°)	− 0.31	− 0.54	0.23
Thoracic bending angle (°)	17.43	14.51	2.92
Lumbar bending angle (°)	10.82	14.44	3.62
Standard deviation lateral deviation (°)	3.76	3.63	0.13
Standard deviation rotation (°)	3.90	3.81	0.09
Kyphosis angle (°)	53.99	60.49	6.50
Lordosis angle (°)	33.40	52.61	19.21
Pelvic distance (mm)	93.85	92.23	1.62
Pelvic height (°)	− 0.01	0.00	0.01
Pelvic height (mm)	0.07	− 0.03	0.10
Pelvic torsion (°)	− 0.20	− 0.72	0.52
Pelvic rotation (°)	− 0.71	0.77	1.48
Scapular distance (mm)	197.70	164.06	33.64
Scapular height (°)	− 0.37	0.15	0.52
Scapular rotation (°)	1.15	1.40	0.25
Left scapular angle (°)	26.13	27.28	1.15
Left scapular angle (°)	28.21	28.53	0.32

Values are taken from this work and research papers^30^.

Age is significantly related to the trunk length D (p ≤ 0.02; rho = − 0.23) and trunk length S (p ≤ 0.001; rho = − 0.27) with a weak effect size. Thus, the trunk length shortens with age, although this correlation could not be established in the younger age groups^26,28^. Possible explanations for a decrease in trunk lengths with age can be seen in the reduced turgor of the intervertebral discs^60^. The intervertebral discs swell less during the unloading phase, resulting in a significantly reduced trunk length that can be measured in total. However, other studies have revealed that a significant reduction in trunk size occurs only after the age of approximately 60 years^43,44^.

The increase in trunk lengths D and S, the thoracic bending angle and the pelvic distance with increasing body height can be explained by the anthropometric standard values of body proportions, i.e. the body parts are in relative proportion to each other^61–63^. For body weight, it can be summarized that the trunk lengths and the distances of the pelvic blades and the scapulae, respectively, increase with increasing body weight, and that there is a greater ventral inclination of the upper body the heavier the subjects are. Similar conclusions can be drawn when looking at the results on BMI. Pelvic and scapular distances and the kyphosis angle increase with increasing BMI, whereas the sagittal trunk tilt decreases with increasing BMI. With increasing body weight or BMI, there is an anterior shift of the body's center of gravity^64^. This ventral pull requires increased effort from the back and shoulder muscles, however, muscle efficiency decreases with age^65^ thus resulting in yielding to the load. This produces in a protracted shoulder position and an increase in the scapula distance^66^. The increasing kyphosis angle of the thoracic spine and ventral trunk tilt are also due to increased weight-related degeneration of the spine and muscle weakness in relation to body mass^67–69^. The fact that only weight and not BMI is positively correlated with the trunk lengths is because taller people, usually, also weigh more^70^.

No group differences can be found with regard to the upper body posture during athletic activity. An explanation of this observation cannot be justified with the current literature. However, the classification of the groups cannot be compared in an optimal way because no distinction was made between the types of sports and the intensity performed in the study. Athletes who play golf once a week, for example, experience different physical demands than swimmers or cyclists.

With regard to the selection of the test subjects, it must be remembered that the assessment of subjective health was carried out by the test subjects themselves, without further mobility tests or a more specific anamnesis with possible scales for the assessment of pain and complaint patterns. However, it can be assumed that all participants can cognitively assess the significance of their statements. An examination by an orthopedic surgeon or another trained specialist could provide additional, objective information about the subjects' state of health. The number of past surgeries and some arthritic joint wear within this age group can also be considered as normal^71^.

When marking the anatomical structures with the reflective markers, palpation of these bone points proved to be more difficult, especially in obese participants, although this was easily overcome as the examiner was experienced. In order to ensure that the recording was always made in the habitual body posture, all participants were asked to perform loosening exercises beforehand. Repeating the scans several times should also reduce possible measurement errors, even though the manufacturer's specification for measurement inaccuracies of the ABW Back Mapper Mobile is already excellent at < 1 mm. The inter- and intra-test reliabilities and validities of video raster stereography are also high^72–75^, so this non-invasive method could be considered an alternative to radiographic examination. This has already been demonstrated in studies of idiopathic scoliosis^76,77^.

The comparison with younger age groups proves on the one hand the changes of the (upper) body posture in the course of life and proves at the same time that the back scanner used here as a topographic measuring method is able to represent and prove these changes. Since the recording of three-dimensional back posture is a fast and radiation-free procedure, it is suitable not only for rehabilitation, but also for preventive diagnostics and follow-up, for example to document the influence of physiotherapeutic therapy or medical training therapy. The comparisons listed between the younger age decades and the present age group describe the changes in upper body posture over the course of life. If these age-specific changes can be correlated with pathologies, it would be possible to improve posture and take preventive action against common complaints through adapted workplace ergonomics, targeted therapies and functional training.

The use of this technique, when taking measurements at different stages of a potentially progressive disease course, would be hugely beneficial as it would allow these stages to be classified by a non-invasive examination method. Overall, these comparisons could help to understand better the development and progression of pathology and, thus, possibly develop an earlier and more targeted therapy whilst the interdisciplinary cooperation would also be simplified.

## Conclusion

Overall, an influence of the upper body posture with regard to the personal, constitutional data (age, height, weight, BMI) can be recognized. However, for the most part, only weak correlations were obtained which is why the clinical influence of these factors occupies such a low position. Thus, the present study made it possible to establish the norm values regarding the upper body posture of a homogeneous, healthy, male subject aged 51–60 years. These data can serve as a reference for future studies on a similar cohort with the same study design.

## Data Availability

The datasets supporting the conclusions of this article are included within the article.

## Abbreviations

BMI: Body mass index
TR: Tolerance range
CI: Confidence interval
